# UV-B and far-red light shape morphological and phytochemical responses in kratom

**DOI:** 10.3389/fpls.2026.1835418

**Published:** 2026-05-29

**Authors:** Mengzi Zhang, Siva Rama Raju Kanumuri, Paul R. Fisher, Brian J. Pearson, Abhisheak Sharma, Christopher R. McCurdy, Jianjun Chen

**Affiliations:** 1 Mid-Florida Research and Education Center, Horticultural Sciences Department, Institute of Food and Agricultural Sciences, University of Florida, Apopka, FL, United States; 2 Department of Pharmaceutics, College of Pharmacy, University of Florida, Gainesville, FL, United States; 3 Horticultural Sciences Department, Institute of Food and Agricultural Sciences, University of Florida, Gainesville, FL, United States; 4 Mid-Columbia Agricultural Research and Extension Center, College of Agricultural Sciences, Oregon State University, Hood River, OR, United States; 5 Department of Medicinal Chemistry, College of Pharmacy, University of Florida, Gainesville, FL, United States

**Keywords:** alkaloid, end-of-day, light quality, *Mitragyna speciosa*, mitragynine, shade avoidance, ultraviolet

## Abstract

**Introduction:**

Kratom (*Mitragyna speciosa*) has gained increasing interest due to its unique leaf alkaloids with pharmacological potential. Comparisons of climate conditions in Florida production areas with those in the plant’s native habitat in Southeast Asia indicate a need to better understand how light quality, particularly ultraviolet (UV)-B radiation, influences plant growth and alkaloid biosynthesis. This study evaluated the effects of far-red (FR) light supplementation and end-of-day (EOD) UV-B exposure on plant growth, photosynthetic performance, and leaf alkaloid content of kratom.

**Methods:**

Plants were grown under controlled-environment conditions under five light treatments: white (W) light alone; W plus 1 or 2 h of EOD UV-B exposure; and W supplemented with FR light combined with 1 or 2 h of EOD UV-B exposure. Total photosynthetic photon flux density (PPFD) was maintained at 300 µmol·m^−2^·s^−1^, with additional supplemental FR and EOD UV-B provided at 90 and 0.3 µmol·m^−2^·s^−1^, respectively. Plant growth, photosynthesis, and alkaloid content were analyzed.

**Results:**

Low-dose EOD UV-B had minimal effects on plant architecture and leaf growth, but slightly reduced leaf biomass and photosynthetic capacity. Extending EOD UV-B exposure from 1 to 2 h reduced the stem caliper by 20%, branching by 14%, and new leaf area by 14%. Supplementation with FR at 30% of total PPFD induced a shade-avoidance response, increasing plant height by 58% to 76% while reducing leaf number, photosynthesis, and biomass. Corynantheidine concentration in leaves increased by 175% under W plus 1 h of EOD UV-B, whereas speciociliatine, mitraciliatine, and isopaynantheine decreased in aging leaves.

**Conclusions:**

Light-quality-induced stress can modify alkaloid accumulation in kratom, and these responses must be carefully balanced against reductions in growth and biomass. These findings improve understanding of environmental regulation of kratom alkaloid biosynthesis and provide a basis for designing lighting strategies to optimize both biomass production and the accumulation of desirable alkaloids.

## 1 Introduction

Kratom (*Mitragyna speciosa*), a member of the family Rubiaceae, is a tropical tree indigenous to Thailand, Malaysia, Myanmar, Indonesia, and other areas of Southeast Asia. Its leaves contain a diverse array of bioactive metabolites, including alkaloids, flavonoids, and terpenoids (Cinosi et al., 2015; Flores-Bocanegra et al., 2020; Chear et al., 2021), which have significant medicinal and pharmacological potential (Martins and Nunez, 2015).

The pharmacological properties of kratom are primarily attributed to several alkaloids. Mitragynine is the most abundant alkaloid, accounting for up to 38.7% of the total alkaloid content in traditional and commercial kratom products and has been reported to produce analgesic effects through non-opioid mechanisms, thereby potentially avoiding some adverse effects commonly associated with classical opioids (Sharma et al., 2019; Eastlack et al., 2020; Obeng et al., 2020; Chear et al., 2021; Sharma and McCurdy, 2021). In contrast, 7-hydroxymitragynine, a potent μ-opioid receptor agonist formed through mitragynine metabolism, is present at very low levels in fresh leaves but may accumulate in processed extracts and is considered a major contributor to increasing kratom’s dependence liability (Ponglux et al., 1994; Kruegel et al., 2016; Lydecker et al., 2016; Yue et al., 2018; Hemby et al., 2019; Singh et al., 2020). Other minor alkaloids, including speciogynine, paynantheine, and speciociliatine, interact with serotonergic and adrenergic receptors and contribute to kratom’s complex pharmacological profile (Obeng et al., 2020; León et al., 2021; Grundmann et al., 2023).

**Figure 1:**
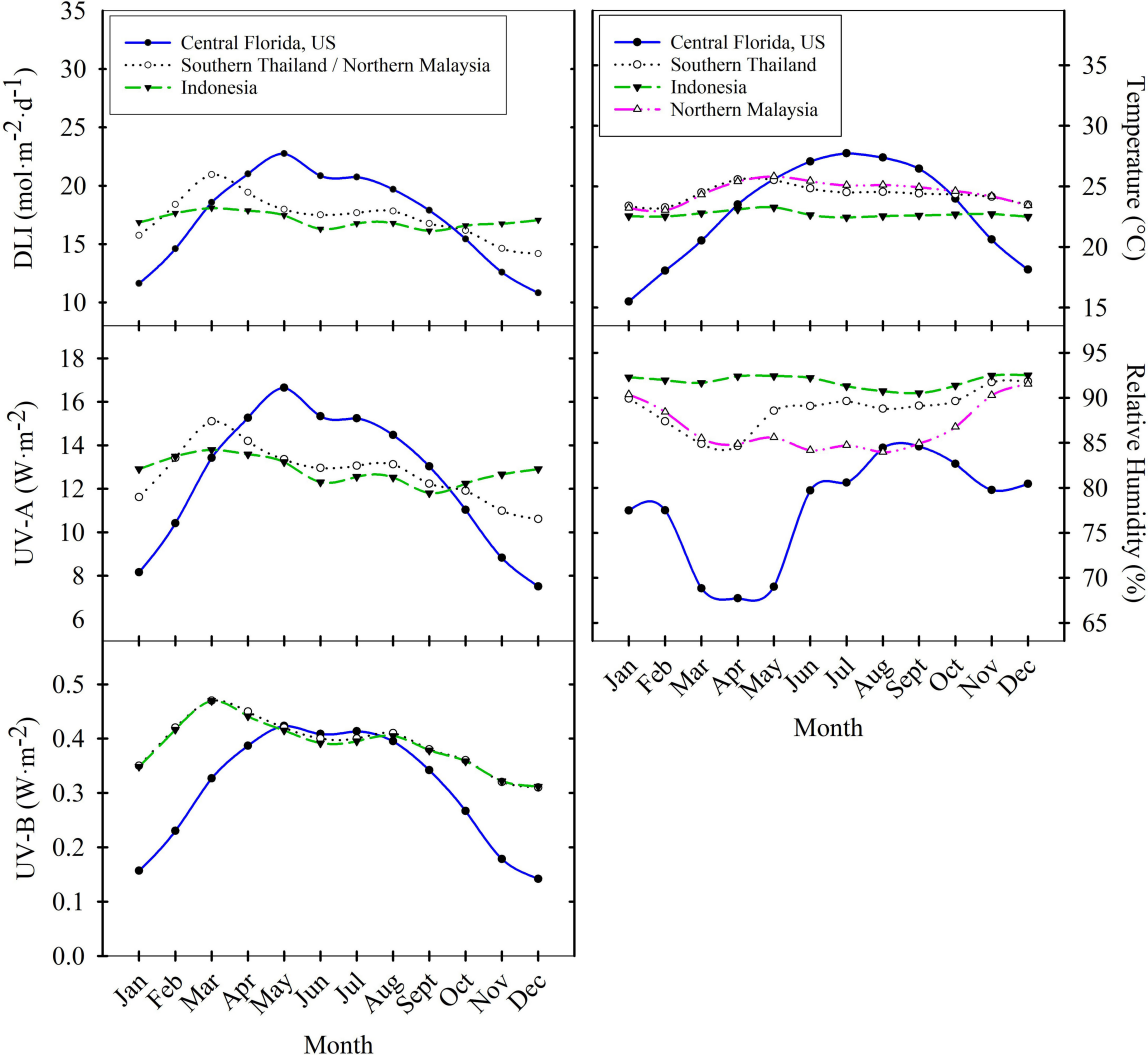
Average daily light integral (DLI), temperature, relative humidity, ultraviolet (UV)-A and UV-B irradiance comparison by month between four locations. Specific locations: Central Florida (Orlando; 28.6064°N, -81.3786°W), Southern Thailand (5.9851°N, 101.3571°W), Northern Malaysia (5.4509°N, 101.5929°W), Indonesia (Borneo; 0.9°N, 114.5°W). Data were drawn from the NASA POWER database.

Given that kratom originates from different tropical regions of Southeast Asia, identifying comparable growing environments is essential for successful cultivation outside its native range (Zhang et al., 2025a). To evaluate the potential to produce kratom of comparable quality in the United States (U.S.), we conducted a climate data analysis in August 2021 using a weather database (NASA Prediction of Worldwide Energy Resources (POWER) Project, 2022) for the period from 2015 to 2020. Four representative locations were selected: Central Florida (Orlando; 28.6064°N, -81.3786°W), Southern Thailand (5.9851°N, 101.3571°W), Northern Malaysia (5.4509°N, 101.5929°W), and Borneo, Indonesia (0.9°N, 114.5°W). While average daily light integral (DLI), temperature, and ultraviolet (UV)-A irradiance were broadly comparable across the four regions (**Table 1**), a marked divergence in ultraviolet B (UV-B) irradiance was observed. Central Florida received about 26% less UV-B radiation than the Southeast Asian locations, despite similar overall light and temperature conditions. Moreover, UV-B exposure in Central Florida exhibited greater seasonality, peaking between May and August and declining substantially during the remaining months (**Figure 1**). In contrast, relative humidity and UV-B irradiance were consistently higher and more stable across Southeast Asian sites (**Table 1**). This persistent reduction and seasonal variability in UV-B exposure under U.S. conditions identify UV-B as a key environmental factor that may constrain kratom growth and secondary metabolite biosynthesis, thereby providing a strong rationale for examining the role of UV-B manipulation in controlled cultivation systems.

**Table 1:** Means (± s.e.) of daily light integral (DLI), temperature, relative humidity, ultraviolet A (UV-A), and ultraviolet B (UV-B) irradiance comparison between four locations from 2015 to 2020.

Location	DLI (mol·m^-2^·d^-1^)	Temperature^z^ (°C)	Relative humidity^z^ (%)	UV-A (W·m^-2^)	UV-B (W·m^-2^)
Central Florida	17.21 ± 1.18	22.88 ± 1.21	77.73 ± 1.73 c	12.45 ± 0.91	0.31 ± 0.03 b
Southern Thailand	17.27 ± 0.56	24.38 ± 0.21	88.76 ± 0.65 ab	12.72 ± 0.38	0.39 ± 0.01 a
Northern Malaysia	17.27 ± 0.56	24.55 ± 0.27	86.76 ± 0.78 b	12.72 ± 0.38	0.39 ± 0.01 a
Indonesia	17.03 ± 0.18	22.7 ± 0.07	91.83 ± 0.20 a	12.84 ± 0.17	0.39 ± 0.01 a

^z^ Measured at 2 meters. Data were drawn from the NASA POWER database. Means sharing the different letters are statistically different by Tukey’s honest significant difference test at P ≤ 0.05. Means without letters are not significantly different. Specific locations: Central Florida (Orlando; 28.6064°N, -81.3786°W), Southern Thailand (5.9851°N, 101.3571°W), Northern Malaysia (5.4509°N, 101.5929°W), Indonesia (Borneo; 0.9°N, 114.5°W). Data were drawn from the NASA POWER database.

Alkaloid biosynthesis is highly responsive to light quality, particularly when exposed to UV radiation (Takshak and Agrawal, 2015; Pandey et al., 2021). UV radiation, which lies at the high-energy end of the solar spectrum, can function as an abiotic stressor. This high-energy radiation often triggers plant defense mechanisms, leading to increased production of secondary metabolites, such as alkaloids. Among the UV spectra, UV-B radiation (280–315 nm) is especially influential because it is readily perceived by plants and strongly associated with stress-induced metabolic reprogramming. Although studies remain limited, UV-B exposure has been shown to stimulate secondary metabolite accumulation by activating stress-related signaling pathways and by regulating transcription of biosynthetic genes (Pandey et al., 2023). Consistent UV-B-induced modulation in indole alkaloid production have been reported in multiple medicinal plant species, including *Clematis terniflora*, *Withania somnifera*, *Coleus forskohlii*, *Zanthoxylum bungeanum*, and *Coleus aromaticus* (Takshak and Agrawal, 2019). Furthermore, in *Catharanthus roseus*, a species with better-characterized alkaloid biosynthesis pathways, UV-B exposure has been shown to enhance indole alkaloid accumulation. For example, Ramani and Jayabaskaran (2008) reported that catharanthine and vindoline concentrations in suspension-cultured cells increased by 3- and 118-fold, respectively, after 48 hours of UV-B irradiation compared to untreated controls. Similarly, Zhu et al. (2015) found that a 1-hour UV-B treatment followed by 72 hours of dark incubation led to substantial increases in strictosidine (528%), ajmalicine (322%), vindoline (20%), and catharanthine (19%). These findings highlight the potential of UV-B radiation to stimulate the production of pharmacologically active compounds in medicinal plants such as kratom.

UV-B radiation can enhance the accumulation of bioactive compounds in many plant species; however, its effects on plant growth are variable. In most studies, UV-B exposure is associated with adverse effects on plant growth and morphology, frequently resulting in reduced stem and petiole elongation and decreased leaf area (Qian et al., 2021). A meta-analysis conducted by Fu and Shen (2017), which synthesized studies published before 2015, found that enhanced UV-B radiation was associated with significant reductions of multiple plant growth parameters, including a 25.6% decrease in height, a 16.4% decrease in basal diameter, and a 46.4% decrease in leaf area index. Biomass accumulation was similarly constrained, with total biomass reduced by 28.2%, belowground by 40.9%, aboveground by 23.3%, fruit biomass by 37.5%, and stem biomass by 23.8%. Similar UV-B-induced reductions in stem length, leaf area, and biomass have been reported across a wide range of species, including soybean (*Glycine max*), maize (*Zea mays*), wheat (*Triticum aestivum*), oats (*Avena sativa*), poplar (*Populus kangdingensis* and *P. cathayana*), pea (*Pisum sativum*), and mulberry (*Morus alba*) (Barnes et al., 1990; Ren et al., 2007; Liu et al., 2013; Chen et al., 2016; Fina et al., 2017; Roro et al., 2017). However, controlled environment studies also demonstrated that supplemental UV-B can stimulate growth under specific conditions. For example, sweet basil (*Ocimum basilicum*) exhibited increases in leaf area, fresh biomass, and dry biomass after seven days of low-dose supplemental UV-B (2–4 kJ·m^−2^·d^−1^) (Sakalauskaite et al., 2012, 2013). Similarly, lettuce (*Lactuca sativa* L.) seedlings showed higher photosynthetic rates and greater relative growth when exposed to supplemental UV-B at field-level intensity (12.5 kJ·m^−2^·d^−1^) (Wargent et al., 2011). Together, these findings suggest that UV-B effects are dose- and species-dependent, underscoring the need for species-specific evaluation.

In contrast to the generally suppressive effects of UV-B radiation, moderate far-red (FR) light has been shown to promote plant growth and photosynthesis by stimulating stem elongation, leaf expansion, and leaf mass accumulation (Park and Runkle, 2017; Zhen and van Iersel, 2017). Supplementing 40 µmol·m^−2^·s^−1^ FR to a red (R) + blue (B) background light increased seedling heights of snapdragon (*Antirrhinum majus*) and zinnia (*Zinnia elegans*) by 64-134% and 52-96%, respectively, compared with plants grown without FR (Zhang et al., 2020). In red leaf lettuce ‘Sunmang’, supplementing 12-148 µmol·m^−2^·s^−1^ FR to a photosynthetic photon flux density (PPFD) of 130 µmol·m^−2^·s^−1^ R+B significantly increased shoot fresh weight by 28-43% and dry weight by 18-30% after 24 days (Lee et al., 2016). Similarly, under a constant PPFD of 160 µmol·m^−2^·s^−1^, FR addition increased total leaf area by 7% and shoot dry weight by 28-50% in geranium (*Pelargonium × hortorum*) and snapdragon seedlings (Park and Runkle, 2017). However, the benefits of FR diminish as proportions increase. Zhen and van Iersel (2017) reported that the quantum yield of PSII increased with FR additions of 0-90 µmol·m^−2^·s^−1^ to red and blue light (PPFD = 200 µmol·m^−2^·s^−1^) but plateaued at ~50 µmol·m^−2^·s^−1^ FR (~25% of total photon flux), suggesting that moderate FR fractions improve photosystem balance while higher fractions provide little additional benefit. Consequently, FR levels around 20% are generally considered the upper beneficial limit for many crop species (Zhen et al., 2021).

As an understory tree native to Southeast Asian rainforests, kratom is adapted to shaded canopy environments enriched in FR radiation, especially during the young plant stage. In tropical forest gaps, R: FR ratios typically range from 0.4 to 0.8, corresponding to FR fractions of approximately 55-70% (Turnbull and Yates, 1993). In addition to these FR-enriched conditions, mature trees, which can reach up to 24 meters in height (Eisenman, 2014), may eventually grow beyond the canopy and escape shade in their natural habitat, becoming exposed to relatively high levels of solar UV-B radiation. Given this ecological background, it is important to understand how kratom growth and secondary metabolism respond to varying light quality stresses. Therefore, the objective of this study was to evaluate the effects of low-dose UV-B and FR-enriched conditions on kratom growth and alkaloid accumulation.

## 2 Materials and methods

### 2.1 Plant material preparation

The study was conducted in two experimental runs, in July and September 2024. In each experimental run, genetically identical *M. speciosa* ‘MR-Malaysian’ kratom plants were selected and grown individually in 11-L containers filled with customized soilless substrate containing 25% Florida peat, 25% Canadian peat, 30% pine bark, and 20% perlite (Reliable Peat Company, Leesburg, FL, USA). Plants were initially established and cultivated under natural light in a greenhouse located in Apopka, Florida, USA (lat. 28˚38’N, long. 81˚33’W), where they were stabilized for three weeks. The average greenhouse temperatures during the first and second experimental runs were 28.6 ± 0.1 °C and 27.5 ± 0.1 °C, respectively. These plants were then transferred to controlled growth rooms for preconditioning.

### 2.2 Growth room setup and environmental control

**Figure 2:**
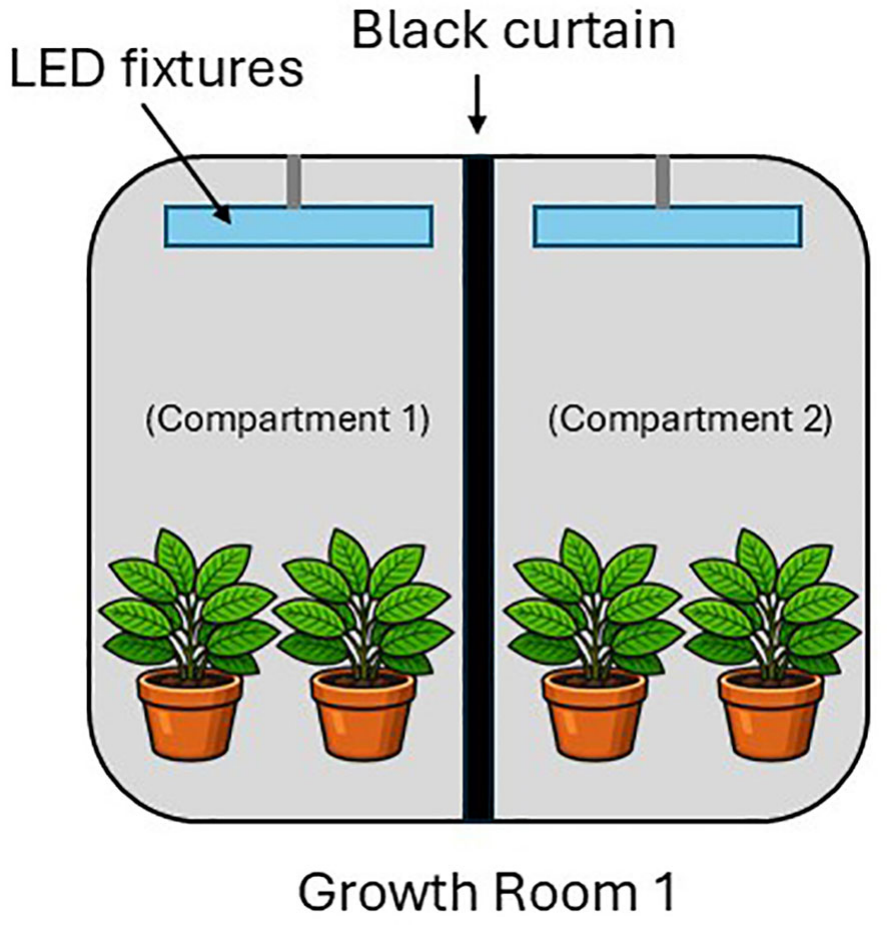
An illustration of the experimental setup in a plant growth room.

The experiment was conducted in five identical indoor growth rooms, each measuring 3 m × 3 m × 3 m, under ambient CO_2_ conditions. Each growth room was further divided into two compartments (**Figure 2**) using black plastic curtains to prevent light contamination between treatments, resulting in a total of 10 compartments. LED fixtures were mounted at the top of the ceiling of each compartment, and the distance between the plant canopy and the LED fixtures was approximately 65 cm. Each compartment contained a metal tilted bench equipped with an automatic irrigation system that irrigates plants daily. Air temperature in all rooms was maintained at 25 °C using air conditioners, with fans operating at a constant high speed to enhance air circulation. Air temperature was adjusted weekly throughout the experimental period, with actual averages reported in **Table 2**. Environmental conditions were continuously monitored using wireless sensors (RXW-THC-900; Onset Computer Corporation, Bourne, MA, USA), and temperature and relative humidity were recorded every 5 minutes via a wireless data logging system (HOBO RX3000; Onset Computer Corporation, Bourne, MA, USA).

**Table 2:** Five light treatments provided by white (W), supplemental far-red (FR), and end-of-day (EOD) ultraviolet-B (UV-B) light-emitting diodes.

Treatment	Light spectrum (µmol·m^−2^·s^−1^)					PPFD (µmol·m^−2^·s^−1^)	FR%	Air temperature (°C)	Relative humidity (%)
	UV-B	B	G	R	FR				
W	0	53.7 ± 1.9	125.2 ± 1.1	116.7 ± 1.2	6.1 ± 1.0	295.8 ± 3.7	2.1 ± 0.3	25.1 ± 0.2	81.8 ± 1.7
W + 1 h EOD UV-B	0.29 ± 0.01	55.8 ± 0.5	126.7 ± 0.4	119.8 ± 0.9	6.3 ± 0.9	302.4 ± 1.0	2.1 ± 0.3	24.7 ± 0.2	81.0 ± 2.0
W + 2 h EOD UV-B	0.29 ± 0.01	53.3 ± 0.7	127.2 ± 0.8	119.3 ± 0.5	5.2 ± 1.1	300.0 ± 0.8	1.7 ± 0.4	25.3 ± 0.3	80.9 ± 1.5
W + FR + 1 h EOD UV-B	0.30 ± 0.01	53.9 ± 0.8	124.1 ± 0.8	119.8 ± 0.7	85.2 ± 5.1	298.1 ± 1.3	28.6 ± 1.8	25.1 ± 0.3	80.5 ± 1.7
W + FR + 2 h EOD UV-B	0.29 ± 0.01	54.8 ± 1.4	125.9 ± 0.8	122.1 ± 1.4	91.6 ± 6.2	303.1 ± 2.0	30.2 ± 1.8	24.9 ± 0.3	78.9 ± 0.4

UV-B, ultraviolet-B (280-315 nm); B, blue (400–499 nm); G, green (500–599 nm); R, red (600–699 nm); FR, far-red (700–799 nm); PPFD, photosynthetic photon flux density.

### 2.3 Preconditioning

Each experimental run lasted six weeks: two weeks of preconditioning followed by four weeks of light treatment exposure.

During preconditioning, the 20 most uniform plants were randomly selected and assigned to 10 compartments, with two plants per compartment, and grown under full-spectrum white (W) light-emitting diodes (LEDs) (VYPR 2p; Fluence Bioengineering, Inc., Austin, TX, USA) for 2 weeks. The PPFD of the W lighting ranged from 298 ± 1 to 301 ± 3 µmol·m^−2^·s^−1^, with a 14-hour photoperiod. Light intensity was measured using a laboratory spectroradiometer (PS-200; Apogee Instruments Inc., Logan, UT) at three representative locations, 140 cm above the bench surface (approximating plant canopy height). Average air temperatures during pre-conditioning ranged from 24.3 ± 0.03 to 24.9 ± 0.02 °C for the first experimental run, and from 24.1 ± 0.03 to 25.1 ± 0.01 °C for the second. To ensure adequate nutrient availability, a moderate rate of 65 g per container of a controlled-release fertilizer (Osmocote Plus 15-9-12, 5–6 months, Scotts, Marysville, OH, USA) was applied at the start of the preconditioning. The fertilizer composition comprises 8.4% ammoniacal nitrogen, 6.6% nitrate nitrogen, 9% P_2_O_5_, 12% K_2_O, 1.3% magnesium, 6% sulfur, 0.5% iron, and trace amounts of boron, copper, manganese, molybdenum, and zinc.

### 2.4 Lighting treatments

**Figure 3:**
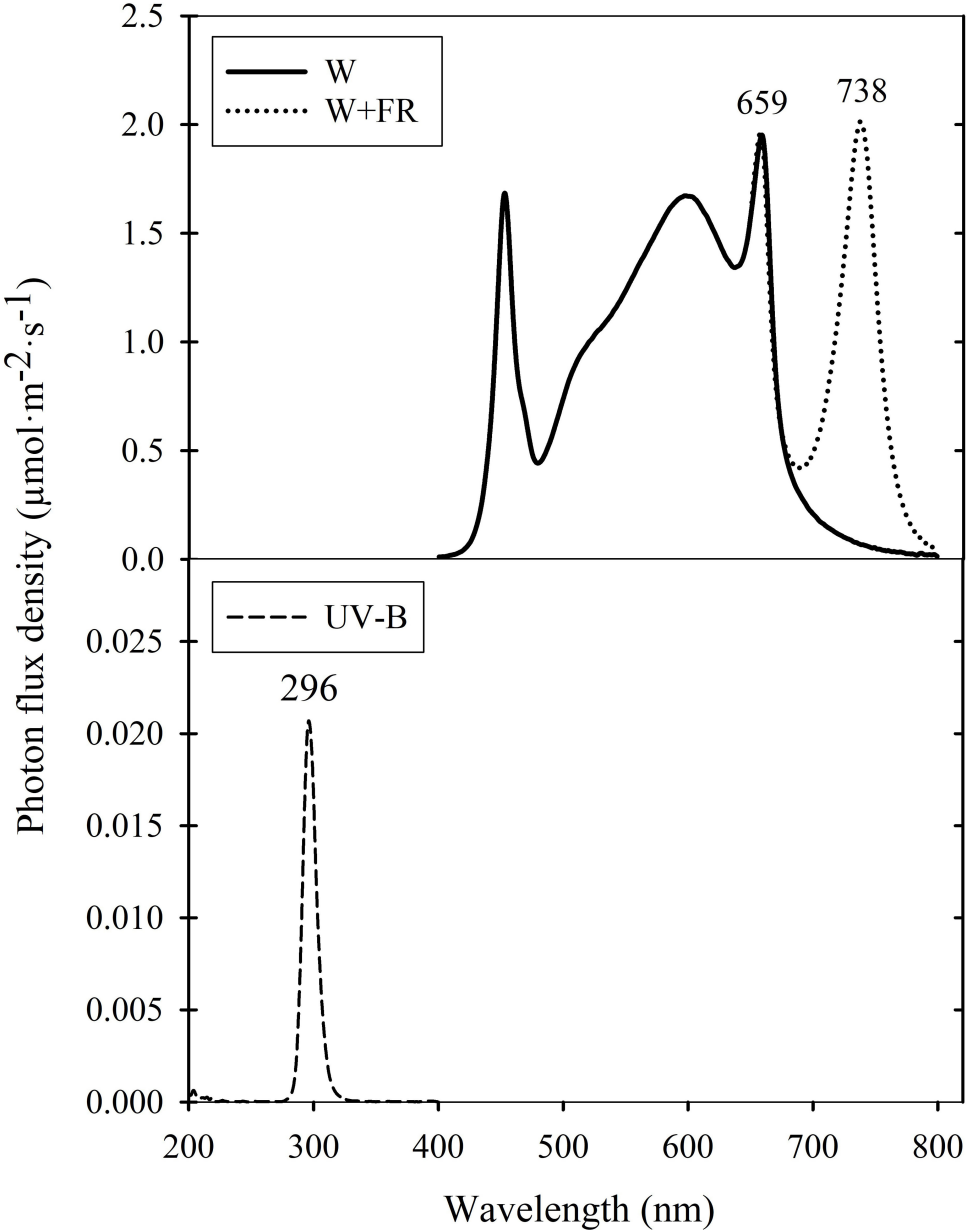
Spectral distribution of white (W) light with or without supplemental far-red (FR; 700–800 nm) and end-of-day ultraviolet-B (UV-B; 280–315 nm) treatments. The total photon flux density was 300 µmol·m^−2^·s^−1^ for W, 390 µmol·m^−2^·s^−1^ for W+FR light (300 µmol·m^−2^·s^−1^ W + 90 µmol·m^−2^·s^−1^ FR), and 0.3 µmol·m^−2^·s^−1^ for UV-B.

After preconditioning, five light treatments were randomly assigned to 10 compartments, with no chamber containing both compartments receiving the same light treatment. Light treatments were re-randomized between the two experimental runs to minimize potential compartment confounding effects. Five light treatments were as follows: (1) W light only; (2) W light followed by 1 h of EOD UV-B radiation (W + 1 h EOD UV-B); (3) W light followed by 2 h of EOD UV-B light (W + 2 h EOD UV-B); (4) W light supplemented with FR light radiation during the photoperiod and followed by 1 h of EOD UV-B light radiation (W + FR + 1 h EOD UV-B); and (5) W light supplemented with FR radiation during the photoperiod and followed by 2 h of EOD UV-B radiation (W + FR + 2 h EOD UV-B). W light was provided by full-spectrum white LEDs at a PPFD of 300 µmol·m^−2^·s^−1^ (DLI = 15.1 mol·m^−2^·d^−1^), with a peak at 659 nm (**Figure 3**). W light consisted of approximately 18% blue (400–499 nm), 42% green (500–599 nm), 40% red (600–699 nm), and 2% far-red (700–799 nm) based on ePPFD (400–800 nm), with detailed photon flux densities provided in **Table 2**. Supplemental FR light was delivered by FR-specific LED fixtures (Ray66 PfrSpec; Fluence Bioengineering, Inc., Austin, TX, USA) emitting a narrow waveband with a peak at 738 nm at a PPFD of 90 µmol·m^−2^·s^−1^ (**Figure 3**), delivering a DLI of 19.7 mol·m^−2^·d^−1^. The 30% FR fraction selected in this study was to approximate understory light conditions while avoiding the extreme spectral imbalance of dense forest shade. Both W and FR lights operated on a 14-hour photoperiod (0600 to 2000 HR) controlled by mechanical timers (BN-LINK Inc., Cucamonga, CA, USA) without ramp-up or ramp-down transitions. EOD UV-B radiation was provided using UV-B lamps (LPB3XUVBS6060/48; Demegrow Inc., Sacramento, CA, USA) at a photon flux density of 0.3 µmol·m^−2^·s^−1^, applied from 2000 to 2100 HR for 1-hour treatments and from 2000 to 2200 HR for 2-hour treatments. Treatments without UV-B were measured using the spectroradiometer standard calibration, whereas treatments with UV-B were measured using a UV-B-specific calibration curve developed by Apogee. All lighting treatments were applied continuously for 4 weeks until the experiment was terminated. A summary of the lighting treatments is presented in **Table 2**.

### 2.5 Plant measurement and data collection

Plant height, canopy width, trunk diameter, branch number, relative chlorophyll concentration (SPAD), and photosynthetic capacity were measured at the beginning and end of the experiment. Plant height was measured weekly from the substrate surface to the primary meristem, and canopy width was recorded at the widest point throughout the experiment. Trunk diameter was measured directly above the substrate surface using a digital fractional caliper (Husky IMS International Ltd., Bolton, Canada). SPAD values were collected using a chlorophyll meter (SPAD-502 Plus, Konica Minolta, Inc., Osaka, Japan) on fully expanded mature leaves located at the 2nd and 3rd nodes from the primary meristem. Three readings were taken per leaf, and the average value was recorded. Photosynthetic parameters, including assimilation rate (*A*), transpiration rate (*E*), intercellular CO_2_ concentration (*Ci*), and stomatal conductance (*gsw*), were measured using a portable gas exchange system (LI-6800; LICOR, Inc., Lincoln, NE, USA). Intrinsic water-use efficiency (*iWUE*) was calculated as *A* divided by *gsw*. Measurements with the LI-6800 were also taken from fully expanded mature leaves at the 2nd and 3rd nodes, with two to three readings per plant. The average of these readings was calculated.

To distinguish newly emerged leaves from existing ones, all leaves exceeding 10 cm in length were marked with a punch hole at the initiation of the experiment. New leaves were defined as those exceeding 10 cm in length at the termination of the study. The number of new leaves per plant was recorded, and their area was measured using a leaf area meter (LI-3000; LICOR, Inc., Lincoln, NE, USA). Fresh weight of the new leaves was recorded using a digital balance (Ranger 3000; Ohaus, Parsippany, NJ, USA), and dry weight was determined after oven-drying the samples at 80 °C for at least five days. Specific leaf area was calculated as new leaf area divided by new leaf dry mass.

### 2.6 Leaf harvest and alkaloid extraction

Leaves from the 2nd and 3rd nodes on both primary and lateral branches were collected at the beginning and end of the experiment. Since kratom leaves grow in opposite pairs at each node, one leaf from each node was collected at the beginning of the study to determine initial alkaloid content, while the corresponding paired leaves were marked and harvested at the end of the study for final alkaloid analysis. Harvested leaves were dried using a forced-air fan-cooling system at ambient room temperature (approximately 23 °C) for seven days, then ground into a fine powder using a commercial grinder (BCG211, KitchenAid, St. Joseph, MI, USA). Alkaloids were extracted and quantified using a validated UPLC-MS/MS method following the protocol described by Zhang et al. (2025a).

### 2.7 Experimental design and data analysis

The experiment consisted of four replications, with two subsamples per replication for each treatment. The two experimental runs were considered as two blocks. Each compartment served as an experimental unit, and each block contained two full sets of treatment replications across 10 compartments, yielding a total of 4 replications (n = 4). Each compartment housed two plants, which were considered as two subsamples. Measurements from subsamples were averaged before statistical analysis. Limited replication was due to the limited availability of plant material, as this species is not commercially available worldwide. Statistical analyses were performed using Mixed Models in JMP Pro 16 (SAS Institute Inc., Cary, NC, USA) with mean separation tests carried out using Fisher’s Least Significant Difference test. Block was considered a random effect, and no significant block effects were detected in any analysis. Statistical tests were considered significant if P ≤ 0.05.

## 3 Results

### 3.1 Changes in plant morphological characteristics

Overall, EOD UV-B had minimal effect on kratom height extension as shown by the comparison between W + EOD UV-B and W alone. In contrast, supplemental FR light increased plant height by 36-64% when comparing W + FR + EOD UV-B with W+ EOD UV-B (**Table 3**). Under a similar comparison, branch formation remained largely unchanged under W with EOD UV-B relative to W alone; however, the addition of FR resulted in an average reduction of 8–10 branches compared to W + EOD UV-B treatments. A similar but more pronounced trend was observed in new leaf production and new leaf area. Supplemental FR resulted in 35-42% fewer new leaves, and 33-36% less new leaf area compared to W + EOD UV-B. Light treatments generally had little effect on canopy width extension, overall plant size (height x width) extension, or trunk caliper growth.

**Table 3:** Means (± s.e.) of plant structural, foliar, and biomass characteristics across five lighting treatments.

Plant Growth Parameters	W	W + 1 h EOD UV-B	W + 2 h EOD UV-B	W + FR + 1 h EOD UV-B	W + FR + 2 h EOD UV-B
Plant Structure					
Height extension (cm)	11.1 ± 1.5 b	11.9 ± 2.2 ab	12.9 ± 2.5 ab	19.5 ± 3.0 a	17.5 ± 5.2 ab
Width extension (cm)	16.6 ± 4.4	16.2 ± 4.7	7.0 ± 4.0	8.6 ± 4.3	9.9 ± 3.9
Height*Width (cm^2^)	203.8 ± 84.8	284.4 ± 102.8	107.9 ± 67.6	188.3 ± 102.8	221.1 ± 102.8
Caliper extension (mm)	1.7 ± 0.5	2.0 ± 0.4	1.6 ± 0.5	1.3 ± 0.5	0.9 ± 0.3
New branch number	22.0 ± 5.0 ab	25.0 ± 4.9 a	21.4 ± 5.0 ab	16.8 ± 5.0 ab	11.3 ± 2.1 b
Leaf Characteristics					
New leaf number	150.1 ± 9.2 ab	184.5 ± 23.0 a	155.5 ± 15.2 ab	120.4 ± 23.0 bc	89.5 ± 17.0 c
New leaf area (cm^2^)	10259.9 ± 631.8 a	10417.4 ± 1334.0 a	8993.9 ± 1028.0 ab	7024.0 ± 1334.0 bc	5754.6 ± 1202.1 c
SPAD start	38.8 ± 1.1	34.5 ± 1.4	38.1 ± 2.0	36.1 ± 2.0	39.3 ± 2.0
SPAD finish	38.0 ± 1.2 a	39.5 ± 2.4 a	37.7 ± 1.5 a	32.6 ± 1.0 b	39.2 ± 1.6 a
Specific leaf area (cm^2^/g)	253.7 ± 14.4	260.3 ± 18.7	256.4 ± 7.8	238.9 ± 6.2	237.5 ± 16.9
Biomass					
New leaf fresh weight (g)	199.8 ± 17.2 a	183.9 ± 24.0 a	168.3 ± 18.6 ab	127.3 ± 24.0 bc	102.3 ± 17.6 c
New leaf dry weight (g)	41.0 ± 4.1 a	39.3 ± 4.6 a	34.8 ± 3.1 ab	29.0 ± 4.6 bc	23.5 ± 3.5 c

W, white; EOD UV-B, end-of-day ultraviolet-B; FR, far-red. Means followed by different letters within a row are significantly different according to Fisher’s LSD test at P < 0.05 (n = 4). Means without letters are not significantly different.

Leaf biomass accumulation followed a pattern similar to plant growth responses (**Table 3**). Under W light, the addition of EOD UV-B, regardless of exposure duration, reduced both fresh and dry weights of newly formed leaves compared to W light alone; however, these differences were not statistically significant. In contrast, W + FR + EOD UV-B treatments significantly decreased new leaf fresh weight by 31-39% and dry weight by 26-32% compared to W + EOD UV-B, with the greatest reductions observed under W + FR + 2 h EOD UV-B, in which most leaf growth parameters were reduced by approximately 32-42% relative to W + 2 h EOD UV-B.

Increasing the duration of EOD UV-B further suppressed kratom caliper, branching, leaf number, and leaf area (**Table 3**). Under W light alone, plants receiving 2 h of EOD UV-B resulted in 20% less stem caliper growth, 14% fewer new branches, 16% fewer new leaves, and 14% less new leaf area compared to those with 1 h of exposure. These duration-dependent reductions were amplified in the presence of FR light. Compared with the W + FR + 1 h EOD UV-B treatment, plants exposed to W + FR + 2 h EOD UV-B exhibited 31% less caliper growth, 33% fewer new branches, 26% fewer new leaves, and 18% less new leaf area.

### 3.2 Changes in photosynthetic parameters

Initial SPAD readings of leaves were comparable, and the readings across treatments by the end of the experiment were also similar, except in plants exposed to W + FR + 1 h EOD UV-B, which had a significantly lower value than the other treatments (**Table 3**). As expected, photosynthetic capacity did not differ among plants at the start of the experiment, as they were acclimated to identical environmental conditions (**Table 4**). By the end of the experiment, plants grown under W light alone exhibited the highest *A*, followed by those receiving W + EOD UV-B, while plants under W + FR + EOD UV-B showed the lowest A. A similar gradient was observed was observed in *E*. Notably, the addition of supplemental FR resulted in a significantly lower *Ci* (by 14%) and a significantly higher *iWUE* (by 73%) compared to W + 2 h EOD UV-B.

**Table 4:** Means (± s.e.) of net assimilation rate (*A*, µmol m^-2^ s^-1^), transpiration rate (*E*, mol m^-2^ s^-1^), intercellular CO_2_ concentration (*Ci*, µmol mol^-1^), stomatal conductance (*gsw*, mol m^-2^ s^-1^), and intrinsic water-use efficiency (*iWUE*, µmol CO_2_ mol^−1^ H_2_O) of plants across five lighting treatments at the initiation and termination of the experiment.

Time of Measurement	Photosynthetic Parameters	W	W + 1 h EOD UV-B	W + 2 h EOD UV-B	W + FR + 1 h EOD UV-B	W + FR + 2 h EOD UV-B
Initiation	*A*	9.9 ± 0.8	10.1 ± 0.7	9.1 ± 0.6	9.9 ± 0.8	10.9 ± 0.6
	*E*	0.0028 ± 0.0005	0.0028 ± 0.0005	0.0019 ± 0.0005	0.0027 ± 0.0007	0.0032 ± 0.0005
	*Ci*	291.4 ± 17.8	292.4 ± 14.8	284.4 ± 7.0	286.5 ± 20.6	300.0 ± 14.4
	*gsw*	0.28 ± 0.06	0.26 ± 0.06	0.18 ± 0.05	0.30 ± 0.08	0.32 ± 0.06
	*iWUE*	38.5 ± 3.9	42.5 ± 7.3	42.4 ± 1.6	36.4 ± 10.3	36.5 ± 4.8
Termination	*A*	11.0 ± 0.4 a	9.9 ± 0.6 ab	9.3 ± 0.5 ab	8.9 ± 0.5 b	9.2 ± 0.5 b
	*E*	0.0036 ± 0.0003 a	0.0030 ± 0.0003 ab	0.0027 ± 0.0003 ab	0.0021 ± 0.0003 bc	0.0016 ± 0.0002 c
	*Ci*	315.1 ± 2.9 a	306.4 ± 9.6 a	306.9 ± 9.6 a	301.0 ± 5.3 a	264.0 ± 9.2 b
	*gsw*	0.37 ± 0.04 a	0.29 ± 0.04 ab	0.25 ± 0.02 bc	0.20 ± 0.02 bc	0.14 ± 0.02 c
	*iWUE*	30.3 ± 2.8 b	38.7 ± 7.1 b	38.5 ± 5.5 b	45.4 ± 3.4 b	66.7 ± 6.6 a

W, white; EOD UV-B, end-of-day ultraviolet-B; FR, far-red. Means followed by different letters within a row are significantly different according to Fisher’s LSD test at P < 0.05 (n = 4). Means without letters are not significantly different.

### 3.3 Changes in concentrations of key bioactive compounds

Light treatments had minimal effects on the concentrations of mitragynine, speciogynine, paynantheine, corynoxine-A, and corynoxine-B at both the initiation and termination of the study (**Table 5**). Additionally, 7-hydroxymitragynine and mitraphylline were below the lower limit of quantification (LLOQ) throughout the study.

**Table 5:** Key alkaloid content (% w/w ± s.e.) in kratom dry leaves at the initiation and termination of the study across five light treatments.

Alkaloids	Time of Measurement	W	W + 1 h EOD UV-B	W + 2 h EOD UV-B	W + FR + 1 h EOD UV-B	W + FR + 2 h EOD UV-B
Mitragynine	Initiation	0.51 ± 0.01	0.50 ± 0.07	0.51 ± 0.05	0.54 ± 0.03	0.61 ± 0.04
	Termination	0.59 ± 0.03	0.56 ± 0.03	0.58 ± 0.07	0.54 ± 0.04	0.54 ± 0.04
7-Hydroxymitragynine	Initiation	BLLOQ	BLLOQ	BLLOQ	BLLOQ	BLLOQ
	Termination	BLLOQ	BLLOQ	BLLOQ	BLLOQ	BLLOQ
Speciogynine	Initiation	0.07 ± 0.01	0.07 ± 0.01	0.07 ± 0.01	0.07 ± 0.01	0.08 ± 0.01
	Termination	0.07 ± 0.01	0.07 ± 0.01	0.07 ± 0.01	0.06 ± 0.01	0.07 ± 0.01
Paynantheine	Initiation	0.10 ± 0.01	0.09 ± 0.01	0.10 ± 0.01	0.11 ± 0.01	0.12 ± 0.01
	Termination	0.11 ± 0.01	0.10 ± 0.01	0.11 ± 0.02	0.10 ± 0.01	0.10 ± 0.01
Corynantheidine	Initiation	0.006 ± 0.001	0.008 ± 0.002	0.005 ± 0.002	0.006 ± 0.001	0.006 ± 0.002
	Termination	0.004 ± 0.001 b	0.011 ± 0.003 a	0.010 ± 0.003 ab	0.010 ± 0.003 ab	0.004 ± 0.001 b
Speciociliatine	Initiation	0.14 ± 0.02 A	0.15 ± 0.01 A	0.13 ± 0.02	0.16 ± 0.02 A	0.11 ± 0.01 A
	Termination	0.07 ± 0.01 B	0.10 ± 0.01 B	0.08 ± 0.02	0.07 ± 0.01 B	0.06 ± 0.01 B
Mitraciliatine	Initiation	0.011 ± 0.002 A	0.013 ± 0.001 A	0.008 ± 0.002	0.013 ± 0.002 A	0.009 ± 0.001
	Termination	0.006 ± 0.001 B	0.008 ± 0.001 B	0.007 ± 0.001	0.007 ± 0.001 B	0.005 ± 0.001
Corynoxine-A	Initiation	0.004 ± 0.001	0.005 ± 0.001	0.004 ± 0.001	0.004 ± 0.001	0.005 ± 0.001
	Termination	0.005 ± 0.001	0.006 ± 0.001	0.005 ± 0.001	0.004 ± 0.001	0.005 ± 0.001
Corynoxine-B	Initiation	0.003 ± 0.001	0.004 ± 0.001	0.002 ± 0.001	0.004 ± 0.001	0.004 ± 0.001
	Termination	0.005 ± 0.001	0.006 ± 0.001	0.005 ± 0.001	0.004 ± 0.001	0.004 ± 0.001
Isopaynantheine	Initiation	0.007 ± 0.001 A	0.008 ± 0.001 A	0.005 ± 0.001	0.008 ± 0.001 A	0.005 ± 0.001
	Termination	0.004 ± 0.001 B	0.005 ± 0.001 B	0.004 ± 0.001	0.004 ± 0.001 B	0.003 ± 0.001
Mitraphylline	Initiation	BLLOQ	BLLOQ	BLLOQ	BLLOQ	BLLOQ
	Termination	BLLOQ	BLLOQ	BLLOQ	BLLOQ	BLLOQ

W, white; EOD UV-B, end-of-day ultraviolet-B; FR, far-red. Mean comparisons were conducted within each alkaloid. Lowercase letters indicate mean separation among lighting treatments within a row; uppercase letters indicate mean separation between experimental stages within a column. Means followed by different letters are significantly different according to Fisher’s LSD test at P < 0.05 (n = 4). Means without letters are not significantly different. BLLOQ=Below Lower Limit of Quantification (limit of quantification = 0.0009% w/w).

Corynantheidine exhibited a clear UV-B-dependent response. Concentrations were comparable among treatments at the beginning of the experiment; however, by the end of the experiment, plants exposed to W + 1 h EOD UV-B showed a significant 175% increase in corynantheidine concentration relative to the W-only control. Longer UV-B exposures followed a similar pattern: W + 2 h EOD UV-B increased corynantheidine concentrations by approximately 1.5-fold compared with W light alone, although this difference was not statistically significant. The addition of FR supplemental had little to slightly negative effects on corynantheidine concentration, but these effects were not statistically significant.

In contrast to the UV-B-induced increase in corynantheidine, three alkaloids, namely speciociliatine, mitraciliatine, and isopaynantheine, were significantly reduced at the end of the experiment under most lighting treatments. Relative to initial concentrations, speciociliatine declined by 33-56%, mitraciliatine by 38-46%, and isopaynantheine by 38-50%. Notably, under treatments involving 2 h of EOD UV-B, alkaloid concentrations generally showed minimal differences between initiation and termination, except for speciociliatine, which decreased by 45% under the W + FR + 2 h EOD UV-B treatment.

## 4 Discussion

UV-B radiation acts as both a stress factor and a signaling cue in plants, with important effects on growth, development, and specialized metabolism. It can inhibit stem elongation, reduce leaf expansion, and induce thicker leaves, while also altering the biosynthesis of secondary metabolites associated with plant protection and environmental adaptation. *M. speciosa*, a woody medicinal plant native to the rainforests of Southeast Asia, produces a range of bioactive compounds, particularly pharmacologically important alkaloids. Despite its increasing scientific and medicinal interest, the effects of light quality on growth and secondary metabolism in this species remain poorly understood. The present study provides the first evaluation of the effects of UV-B and FR light on plant growth, physiological performance, and bioactive compound accumulation in *M. speciosa*.

### 4.1 Low-dose UV-B had minimal effects on plant growth

This study showed that EOD UV-B treatments did not significantly reduce kratom growth or photosynthetic performance. Although net assimilation rate, transpiration, and stomatal conductance were relatively lower under EOD UV-B than under W light alone, these changes appeared to be insufficient to cause significant reductions in plant growth or biomass accumulation.

The minimal growth response observed in this study may be attributable to the relatively low UV-B dose applied, which likely remained below the threshold required to inhibit growth in kratom. Treatment intensity and duration were selected based on both preliminary experiments and natural UV-B exposure under sunlight. In pilot experiments (unpublished data), plants exposed to 0.5 µmol·m^−2^·s^−1^ UV-B for 14 h developed visible leaf damage, whereas the lower intensity and shorter exposure used here (0.3 µmol·m^−2^·s^−1^) were intended to impose stress without causing severe injury. This treatment was also within a biologically realistic range, given that UV-B accounts for about 5% of solar radiation reaching the Earth’s surface and typically delivers 2 to 12 kJ·m^−2^·day^−1^ during the cropping season (UNEP, 2002). Converted to photon flux at 296 nm, near the peak emission of our UV-B lamp, this corresponds to an instantaneous intensity of approximately 0.4 µmol·m^−2^·s^−1^, depending on daily exposure duration. Therefore, the UV-B treatment applied in this study was likely too mild to produce a measurable reduction in growth.

Plants vary in their sensitivity to UV-B, and responses differ across species. Similar to our findings, Liu et al. (2023) reported that a daily UV-B dose of 15.55 kJ m^−2^ day^−1^ (≈ 0.45 µmol m^−2^ s^−1^ instantaneous) did not alter shoot fresh or dry mass in lettuce. In contrast, Xiao et al. (2023) found that a much lower dose of 2.81 kJ·m^−2^·day^−1^ (≈ 0.08 µmol·m^−2^·s^−1^) increased leaf area, branch number, and biomass in *Eucommia ulmoides*. Conversely, several studies have reported growth inhibition even at relatively low UV-B doses. For example, Mariz-Ponte et al. (2019) observed that a daily UV-B dose of only 0.353 kJ·m^−2^·day^−1^ significantly reduced tomato growth and biomass accumulation. In Arabidopsis thaliana, UV-B fluence rates as low as 0.1 µmol·m^−2^·s^−1^ can activate UV-B-specific signaling pathways (O’Hara et al., 2019). Given that our dose of 0.9 kJ·m^−2^·day^−1^ did not significantly affect kratom biomass accumulation, this suggests that kratom is comparatively tolerant of UV-B radiation.

Taken together, our results suggest that the UV-B threshold negatively affecting kratom growth lies between 0.9 kJ·m^−2^·day^−1^ (0.3 µmol·m^−2^·s^−1^ for 2 h, as used in this study) and 10.2 kJ·m^−2^·day^−1^ (0.5 µmol·m^−2^·s^−1^ for 14 h, based on unpublished preliminary trials). These findings further indicate that cumulative daily UV-B exposure may be more critical for growth inhibition than instantaneous UV-B intensity alone. Additional studies applying higher UV-B doses for shorter durations or similar doses over longer exposure periods are needed to more precisely define the UV-B sensitivity threshold for kratom growth and development.

### 4.2 FR at 30% adversely affected plant growth

Our results further indicated that, despite being an understory species, kratom responded negatively to supplementation with 90 µmol·m^−2^·s^−1^ FR light (30% FR fraction). Growth suppression was most evident in reduced leaf production and expansion, and lower overall biomass accumulation. The decline in leaf biomass was primarily attributable to fewer leaves with smaller leaf area, which likely reduced canopy light interception and subsequent carbon assimilation. These morphological changes were accompanied by lower photosynthetic capacity. Physiologically, FR supplementation reduced stomatal conductance and transpiration by 31-44% and 30-41%, respectively, compared with treatments without FR. Given the essential roles of stomata in CO_2_ uptake and transpiration, the reductions in *gsw* and *E* likely contributed to lower *A* and suppressed plant growth (Wang and Chen, 2003; Wang et al., 2026). In addition, plants exposed to W + FR + 2 h EOD UV-B showed significantly lower *Ci* and higher *iWUE*. Collectively, these responses indicate that FR-induced stomatal closure restricted CO_2_ diffusion into the mesophyll and reduced water loss, thereby limiting photosynthetic performance and contributing to reduced biomass accumulation.

Plants typically respond to shade stress, or a low R:FR ratio (higher FR fraction), through either shade-tolerance or shade-avoidance strategies (Gommers et al., 2013; Jeong et al., 2024). Shade tolerance is characterized by increased leaf expansion and reduced leaf thickness, whereas shade avoidance involves stem or petiole elongation, reduced branching, and diminished investment in leaf biomass. In this study, kratom exhibited clear shade-avoidance responses under FR supplementation at a total PPFD of 300 µmol·m^−2^·s^−1^, including increased plant height, reduced branching, limited leaf expansion, and lower leaf biomass. Such responses are typical of plants exposed to strong shade cues, where carbon allocation is preferentially directed toward vertical growth at the expense of biomass accumulation (Umesh et al., 2023).

The combination of relatively low PPFD and a high FR fraction in this study simulated a light environment similar to that of dense tropical understories in kratom’s natural habitat, where light is both limited and enriched in FR. Under these conditions, young kratom plants may activate shade-avoidance responses as an adaptive strategy to enhance vertical growth and improve access to light. This interpretation is ecologically consistent with kratom’s natural growth habit, as mature kratom trees can reach heights of up to 25 m in their native environments (Eisenman, 2014), allowing them to eventually escape understory shading.

Interestingly, in a separate study, kratom exhibited a shade-tolerant response, including enhanced leaf production, leaf expansion, and biomass accumulation under greenhouse shade cloth conditions compared with unshaded greenhouse conditions (Zhang et al., 2022). However, the specific light spectrum and FR percentage under the shade treatment were not quantified at the time, and a different kratom cultivar was used. Collectively, these findings may suggest that both FR percentage and cultivar selection should be carefully considered and further investigated to better predict kratom growth and developmental responses to shade-enriched production environments.

UV-B and FR share overlapping signaling components that regulate shade responses, often in antagonistic ways (Fraser et al., 2016; Yadav et al., 2020). UV-B perception by the photoreceptor UVR8 leads to direct interaction with CONSTITUTIVE PHOTOMORPHOGENIC 1 (COP1), promoting ELONGATED HYPOCOTYL 5 (HY5) accumulation and suppressing shade-avoidance traits such as stem elongation. In contrast, FR is primarily perceived by phytochromes, particularly phyA, which modulates the stability of the COP1/SPA complex and ultimately promotes HY5-mediated shade-avoidance responses under low R: FR conditions. As a result, UV-B and FR act antagonistically in regulating shade-related growth responses. Because both high FR fractions and UV-B exposure can independently reduce biomass accumulation, disentangling their relative contributions under combined W + FR + EOD UV-B treatments is challenging. However, the overall enhanced stem elongation observed under FR supplementation, in contrast to the typical UV-B response, suggests that FR, likely due to its higher intensity, may be the primary driver of the growth patterns observed in this study. Future studies using higher UV-B doses in combination with lower FR fractions will be necessary to more clearly resolve the interactive effects of these light signals on kratom growth and development.

### 4.3 Alternation of bioactive compounds by UV-B and FR

A defining characteristic of *M. speciosa* is its production of diverse monoterpene indole alkaloids (MIAs), which arise from the convergence of the shikimate-derived tryptamine pathway and the monoterpenoid secoiridoid pathway (Schotte et al., 2023; Zhang et al., 2025b). UV-B is a well-established regulator of specialized metabolism and has been shown to enhance alkaloid biosynthesis in medicinal plants by increasing precursor availability and stimulating expression of genes involved in MIA formation (Zhu et al., 2015; Wang et al., 2025; Wu et al., 2025).

**Figure 4:**
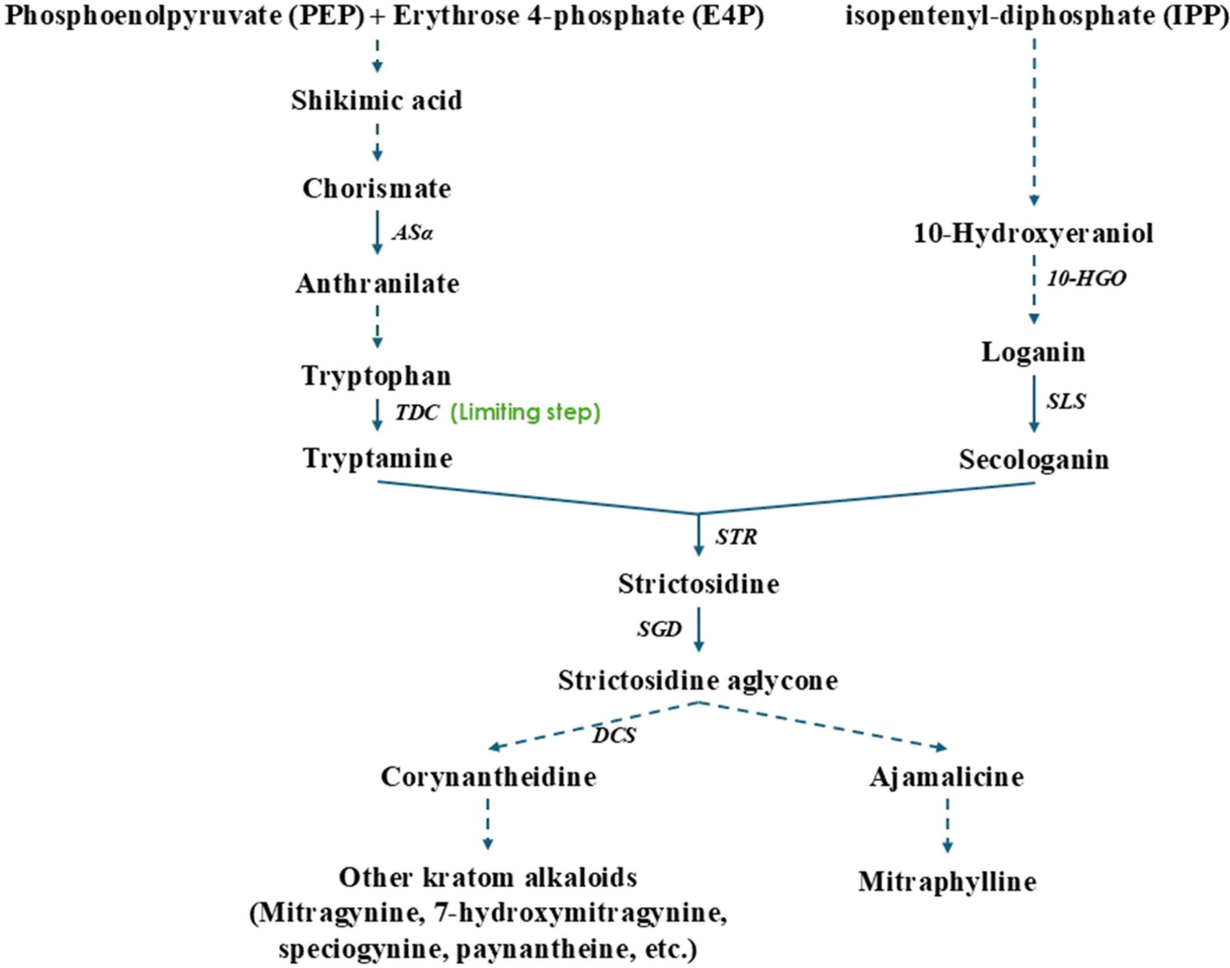
A simplified hypothetical kratom alkaloid biosynthesis pathway compiled from multiple studies (Charoonratana et al., 2013; Zhu et al., 2014; Pan et al., 2016; Kim et al., 2023; Schotte et al., 2023; Ramos-Valdivia and Cerda-García-Rojas, 2024; Laforest et al., 2025).

Among the alkaloids measured in this study, corynantheidine was the most responsive to EOD UV-B. Its concentration increased significantly under W + 1 h EOD UV-B, W + 2 h EOD UV-B, and W + FR + 1 h EOD UV-B. Based on current kratom biosynthetic models (**Figure 4**) (Charoonratana et al., 2013; Zhu et al., 2014; Pan et al., 2016; Kim et al., 2023; Schotte et al., 2023; Ramos-Valdivia and Cerda-García-Rojas, 2024), corynantheidine occupies an important position in the network leading to mitragynine-related alkaloids. Upstream of this step, strictosidine is formed from tryptamine and secologanin and serves as the central precursor for MIA biosynthesis. The increased corynantheidine concentration observed here, therefore, suggests greater flux through the early stages of alkaloid biosynthesis. Although this response does not demonstrate parallel increases in all downstream alkaloids, it supports the view that UV-B promoted corynantheidine formation and may have stimulated the broader kratom alkaloid pathway.

This interpretation is consistent with precursor-feeding studies. Charoonratana et al. (2013) showed that supplying both tryptamine and loganin increased mitragynine accumulation in kratom leaves, whereas loganin alone did not. This finding indicates that precursor availability, especially tryptamine supply, may constrain pathway output. Similar UV-B responses have been reported in *Catharanthus roseus*, in which UV-B increased expression of several key biosynthetic genes encoding tryptophan decarboxylase, 10-hydroxygeraniol oxidoreductase, secologanin synthase, and strictosidine synthase (Zhu et al., 2015). Comparable transcriptional regulation has not yet been demonstrated in kratom. However, the increase in corynantheidine under EOD UV-B is consistent with enhanced activity in early biosynthetic steps, possibly through increased precursor production or upregulation of genes upstream of strictosidine formation. Verification of this mechanism will require targeted analyses of gene expression and enzyme activity. A recent study on the *de novo* production of mitraygynine and speciogynine in *Saccaromyces cerevisiae* showed that numerous shunt products derived from the activity of strictosidine synthase and dihydrocorynantheine synthase, highlighting them as candidates for enzyme engineering to further improve kratom MIAs production in yeast (Holtz et al., 2024).

In contrast, speciociliatine, mitraciliatine, and isopaynantheine declined significantly over the course of the experiment, with the largest reductions occurring under W light alone and under short EOD UV-B exposure. Under the same treatments, mitragynine increased only slightly and not significantly. These patterns indicate that alkaloid composition was influenced not only by light treatment but also by leaf developmental stage during the 4-week study. This interpretation agrees with Laforest et al. (2023), who showed that speciociliatine predominates in juvenile leaves and decreases with maturation, whereas mitragynine becomes more abundant in mature leaves. The decreases in speciociliatine and mitraciliatine reported in that study closely parallel the trends observed here.

Differences in alkaloid stereochemistry may also contribute to these developmental patterns. Speciociliatine, mitraciliatine, and isopaynantheine are 3R isomers, whereas mitragynine is a 3S isomer. Taken together, the present results and those of Laforest et al. (2023) suggest that kratom leaves undergo a developmental shift in alkaloid composition, with younger leaves tending to accumulate more 3R compounds and older leaves tending to accumulate more 3S compounds. Thus, the declines in speciociliatine, mitraciliatine, and isopaynantheine observed here were likely driven largely by ontogenic changes, although some contribution of light treatment cannot be ruled out.

## 5 Conclusion

This study systematically evaluated the effects of light-related stress, specifically FR supplementation and EOD UV-B, on growth and alkaloid biosynthesis in kratom, an emerging medicinal plant of increasing importance. Low-dose EOD UV-B exposure (0.4-0.9 kJ·m^−2^·day^−1^) had minimal effects on photosynthesis, growth, and development, but significantly increased corynantheidine accumulation. These results suggest that parts of the alkaloid biosynthetic pathway are particularly responsive to UV-B, and that optimizing UV-B intensity and exposure duration may offer a practical strategy to enhance the production of desirable bioactive compounds in kratom. By contrast, supplementation with a high FR fraction (30%) under a total PPFD of 300 µmol·m^−2^·s^−1^ induced pronounced shade-avoidance responses, including stem elongation, reduced leaf growth and expansion, lower biomass accumulation, and reduced photosynthetic capacity. Thus, FR supplementation under these conditions is unlikely to benefit kratom production or improve the accumulation of target bioactive compounds. The observed declines in speciociliatine, mitraciliatine, and isopaynantheine over time further suggest that alkaloid accumulation is developmentally regulated. Collectively, these findings advance understanding of environmental regulation of kratom alkaloid biosynthesis and provide a basis for refining lighting strategies to optimize both biomass production and alkaloid profiles.

## Data Availability

The raw data supporting the conclusions of this article will be made available by the authors, without undue reservation.
